# And Now Comes Waterville

**Published:** 1889-05

**Authors:** 


### HALL'S
### JOURNAL OF HEALTH

TRUTH DEMANDS NO SACRIFICE: ERROR CAN MAKE NONE.

VOL. 36. MAY, 1889. No 5.

### AND NOW COMES WATERVILLE.

Y. M. C. A. ROOMS, WATERVILLE, N. Y.

SIRS: Please discontinue the Journal now being sent to our address: if there is anything due, please send bill to C. F. Carpenter, Treasurer, Waterville, Me.

Yours,

April 10th, 1889. E. A. PIERCE, General Secretary.

We have heretofore had occasion to remark that we do not publish HALL'S JOURNAL OF HEALTH in the interest of any theological system or order of belief, nor shall we be deterred from publishing any facts of interest which we know to be true, because they serve to weaken or destroy somebody's faith in any of the thirty-nine articles which, in the sixteenth century constituted the sum and substance of the Protestant religion, and which, doubtless, the above named organization feels it incumbent upon itself to sustain and promulgate, even in this outgoing nineteenth century.

It has always been the policy of the church to keep its laity in ignorance of every natural, historical, or theological truth which in anywise controverts its established creed. It was so in the time of Socrates under a Pagan system which disputed the immortality of the soul.

It was so in the days of Galileo and Servetus, when it was heresy to deny that the earth was the centre of our planetary system, stationary in the heavens.

It was so three years ago when Bishop Potter cautioned the Rev. R. Heber Newton, of his Diocese, against giving his congregation the true history of that remarkable collection of ancient writings, historical, poetical and allegorical, included in the "Old Testament," and claimed by the church, as a kind of religious politics, to have been produced under the direct inspiration of the Almighty, word, and line, and precept from beginning to end.

It is so to-day with certain fossilized associations, whose corner stone is the ignorance of past ages, and whose edifice the conglomerated myths, fables, dogmas and superstitions which have been reverently nursed and tended, and kept alive by a supersensual sacerdotalism, which denies to reason her rights, to philosophy her evidences, to science her conclusions and to nature her everlasting records.

We do not mean, we have never meant to be irreligious in any true conception of the term, and if we have sometimes given place to well established facts and occurrences, which *prove* that the Young Men's Christian Association of Waterville is right in teaching the accountability of man for "deeds done in the body," in the great hereafter into which he must pass, a disembodied spirit, with a continuance of life which reaches far into futurity, even without end ; the association ought to join hands with us rather than shut its doors against us, because forsooth we decline to accept as our God, the ideal Jehovah, a man created, man dominated deity, reflecting the desires, prejudices, hopes and passions of that ever persistent race, but never once pointing to a life hereafter. Such a God answered the requirements of the Jewish leaders of tribes, because he was always on their side, sanctioning their worst offences, and freely and magnanimously acquitting them of their crimes, whenever they became too flagrant for human endurance ; but he was never fitted to survive the ignorance, superstition and folly of those bygone days.

It is in great measure, the incorporation of these barren ideas into the tenets of modern Christianity, that has driven so many reasoning minds from the Christian fold, even into materialism and agnosticism—for it is far better to affirm that "we do not know," than to assume to know irreconcilable, inconsistent and impossible things—to a belief in which both human reason and human justice revolt

We have only the kindest feelings towards the various Young Men's Christian Associations of whatever denominational cast. As a rule they do great good, and we are glad to be able to conscientiously labor in the same field. It is not essential to moral rectitude that we should harmonize upon those matters, which belong exclusively to faith. If we hold that the attributes of the Universal Father are the opposite of jealousy, hatred, cruelty and revenge, are we to be ostracised for it by our Christian brethren ? If we assert and prove by witnesses as numerous as the stars, that the immortality of the soul is established by the return of spirits to earth, and their intelligent communication with mortals, is it an offense against religion ? Even if we differ in this from the directors of Young Men's Christian Associations, can we not work together against the materialistic tendency of the age ?

"Shall I ask the brave soldier who fights by my side
In the cause of mankind, if our creeds agree ?"

A distinguished agnostic in the April number of the North American Review, gives utterance to the following statement.

"No person has come back from the unseen world. No authentic message has been delivered. Through all the centuries, not one whisper has broken the silence that is beyond the grave. Countless millions have sought for some evidence, have listened in vain for some word."

Against this we have the counter sentiment of a no less renowned author, Mrs. Sarah C. Hall, in these words. "I am as sure that 'the dead'—wrongly so called—can and do communicate with the 'living,' as I am that my right hand holds the pen with which I write."

These opposite statements unsustained by proof, are of equal weight, but fortunately for the truth the evidences of spirit communion with mortals in the past, are as authentic as any other accepted fact of history. In our day, they are within the reach of every honest investigator.

No one who assumes to instruct mankind has the right to shut his eyes, stop his ears, and fold his arms inertly, and say "I have no proof. I don't know." Does he know there is such a continent as Africa, such a country as China ? Has he been there ? Does he really know by the same token that he knows that he don't know that the soul is immortal, that the spirits of mortals in the after life do communicate with incarnated spirits here ? Let us be fair and reasonable in all things. There is indeed no fact in the present, concerning which there is such an abundance of affirmative testimony, so varied and wide spread and yet in such perfect accord, as that of spirit return, which disposes of the whole question.

We have heretofore presented a number of such facts as conducive to good health of both body and mind, and shall continue to do so in future, and one of our chief regrets is that the Young Men's Christian Association of Waterville Me., arrays itself in opposition to them. Does it also spurn the Bible evidences of this sublime reality ? They are very frequent and positive. Must the Bible and HALL'S JOURNAL be tabooed its reading room, because they agree upon this vital question ?

We do not publish the Journal with a view to its acceptance by any one class, nor for worldy gain, but to do good. When we fail in this we shall be ready to lay down our pen.

